# Trends in Smoking and Flavored Tobacco Use in California: Black–White Disparities, 2003–2023

**DOI:** 10.31586/gjeid.2025.6202

**Published:** 2025-10-21

**Authors:** Shervin Assari, Babak Najand, John Ashley Pallera, Ali Farhoudian

**Affiliations:** 1 Department of Internal Medicine, Charles R Drew University of Medicine and Science, Los Angeles, CA, USA; 2 Department of Psychiatry, Charles R Drew University of Medicine and Science, Los Angeles, CA, USA; 3 Department of Public Health, Charles R Drew University of Medicine and Science, Los Angeles, CA, USA; 4 Marginalization-related Diminished Returns (MDRs), Los Angeles, CA, USA; 5 Charles R Drew University of Medicine and Science, Los Angeles, CA, USA; 6 Department of Psychiatry, School of Medicine, Tehran University of Medical Sciences, Tehran, Iran

**Keywords:** California, tobacco use, smoking trends, flavored tobacco, menthol, race, racial disparities, Black adults, White adults, health equity, tobacco control, California Health Interview Survey (CHIS), Interrupted Time Series Analysis, healthcare Disparities, Ethnic and Racial Minorities, Black people, White people

## Abstract

**Background:**

Tobacco control policies nationwide have contributed to a substantial decline in cigarette and tobacco use, with particularly sharp reductions observed in states such as California that have implemented restrictive bans, strong prevention measures, and high excise taxes. While these policies have led to overall decreases in tobacco use, progress has not necessarily been distributed equally across racial groups. Understanding long-term trends by race is critical for addressing equity gaps in tobacco prevention and control. Evidence suggests that some racialized groups may experience slower or delayed declines, raising concerns about equity in public health gains.

**Methods:**

We analyzed data from the California Health Interview Survey (CHIS) spanning 2003–2023. Trends in current smoking were examined separately for non-Latino Black and non-Latino White adults. We also assessed current use of flavored tobacco products, given California’s statewide ban enacted in 2021. Changes were evaluated in both absolute terms (percentage point declines) and relative terms (percent reduction from baseline).

**Results:**

Smoking prevalence declined from 17.2% in 2003 to 5.2% in 2023 among White adults and from 19.9% to 9.0% among Black adults. This represents a 12.0 percentage point (69.8%) decline for Whites compared with a 10.9 percentage point (54.8%) decline for Blacks. For flavored tobacco use, prevalence decreased from 8.0% to 4.7% among White adults but only from 11.9% to 10.8% among Black adults. This corresponds to a 3.3 percentage point (41.3%) decline for Whites compared with a 1.1 percentage point (9.2%) decline for Blacks.

**Conclusions:**

Although both Black and White adults in California experienced reductions in smoking over the past two decades, White adults showed larger declines in both absolute and relative terms. Disparities were even more pronounced for flavored tobacco use, where declines were minimal among Black adults despite the statewide ban. These findings suggest that Black populations in California may have been left behind by tobacco control progress, especially regarding flavored products. Given the history of targeted marketing by the tobacco industry, the role of flavors in increasing dependence, and reduced access to cessation resources in Black communities, targeted policies and culturally tailored interventions are needed to ensure equitable reductions in tobacco use. Greater attention to flavored tobacco in Black communities may help narrow these disparities and advance California’s tobacco endgame goals.

## 1. Introduction

Cigarette smoking among U.S. adults has fallen dramatically, dropping from over 40% in the 1960s to around 12–13% by 2020 [1-3], but the pace of decline has not been uniform across racial and ethnic groups [4-6]. From 2011 to 2020, smoking prevalence declined from 20.6% to 13.3% among non-Hispanic White adults and from 19.4% to 14.4% among non-Hispanic Black adults [5]. Despite this, relative disparities remained largely unchanged, indicating persistent equity gaps in tobacco control gains [7]. Additional national analyses show that smoking declines have been slower among African Americans groups compared to Whites, especially when intersecting with lower educational attainment [8]. A recent U.S. Surgeon General’s advisory emphasizes that despite overall declines, progress in smoking cessation continues to be uneven across racial, ethnic, socioeconomic, and other demographic lines [9].

In many countries where tobacco use is declining overall, reductions are disproportionately benefiting more advantaged groups, thereby widening health inequities [10-12]. For instance, in New Zealand, although adult smoking has decreased, Indigenous Māori and Pacific peoples continue to have significantly higher prevalence than European/Other populations, underscoring enduring disparities [13-16]. Research across high-income settings likewise suggests that many tobacco control measures (e.g., media campaigns, smoke-free policies) tend to reach higher socioeconomic status populations more effectively, which can inadvertently worsen inequalities even as the overall prevalence falls [17].

There is limited evidence of strong differential effects of smoking policies and restrictions in workplaces and public places, although individuals in higher occupational groups may be somewhat more likely to shift their attitudes or behaviors in response to such policies. School-based smoking restrictions may have greater impact among girls, while restrictions on sales to minors appear to be more effective for girls and younger children. Increasing tobacco prices may reduce smoking more among lower-income adults and those in manual occupations, though some findings suggest that adults with higher education may also be particularly sensitive to price changes [18-22]. Price increases also appear to influence youth under age 25, with some evidence indicating that boys and non-White youth may be especially price-sensitive [23].

In California, repeated cross-sectional data like the California Health Interview Survey (CHIS) [24-27] document impressive overall declines in adult smoking since the early 2000s, yet persistent disparities across racial/ethnic groups appear to remain. Reports highlight that flavored tobacco, more heavily marketed to and used by Black and other marginalized communities, still contributes to unequal reductions [28]. While many subgroups have benefited from policy changes, Black adults in California have often experienced slower declines in flavored product use, raising concerns of inequitable public health impact [29-32].

Together, these findings suggest that even as tobacco control successes reduce use overall, structural and social determinants including targeted marketing, unequal access to cessation resources, and the diminished returns of socioeconomic gains contribute to uneven progress [33]. This underscores our need to examine trends in current smoking and flavored tobacco use among Black and White adults in California, to inform policies aiming for equity, not just overall reductions.

## 2. Aim

Building on this evidence, the present study aimed to examine long-term trends in current smoking and flavored tobacco product (FTP) use among non-Latino Black and non-Latino White adults in California, using data from the California Health Interview Survey (CHIS) [34-36]. While overall smoking rates have fallen substantially over past decades, some research suggests that these declines may have not been shared equally across racial and socioeconomic groups. Black adults, who are being disproportionately targeted by tobacco marketing and face greater structural barriers to cessation, may have experienced slower declines in both smoking and flavored product use. By directly comparing patterns of decline in both absolute and relative terms between Black and White adults, this study sought to assess whether existing tobacco control policies in California have reached and impacted both racial groups equitably. Understanding these patterns may help guide future policy efforts toward closing persistent racial gaps in tobacco use, rather than allowing progress to primarily benefit more advantaged populations.

## 3. Methods

### 3.1. Design and Setting

We conducted a secondary analysis of publicly available data from the California Health Interview Survey (CHIS) [24-27], a repeated cross-sectional survey designed to provide representative estimates of the health of California’s population. CHIS is the largest state health survey in the United States and uses a multistage sampling design to capture diverse sociodemographic groups across California.

### 3.2. Data Source: CHIS

We used combined CHIS survey years from 2003 through 2023. CHIS data are collected through random sampling strategy in multiple languages and weighted to be representative of the non-institutionalized California population [34-36].

### 3.3. Study Population and Eligibility

Analyses were restricted to adults aged 18 years or older who self-identified as non-Latino Black or non-Latino White. Other racial/ethnic groups were excluded to allow a focused comparison between Black and White adults.

### 3.4. Variables

Two outcomes were examined: (1) current smoking, defined as self-reported smoking of cigarettes during the last 30 days [37,38] and (2) current flavored tobacco use, defined as use of any flavored tobacco product within the past 30 days [39-41] (survey month). Demographic covariates were not included in this descriptive trend analysis, as the primary aim was to compare overall changes between groups over time. Race was self-identified by the participant and was either Black/African American or non-Latino White.

### 3.5. Statistical Analysis

We calculated prevalence estimates for current smoking and flavored tobacco use in each available CHIS year. To describe long-term changes, we focused on the earliest available year (2003) and the most recent year (2023). Prevalence estimates were presented side-by-side for Black and White adults. To evaluate changes in smoking prevalence over time, we calculated declines in two complementary ways. Absolute decline was measured as the difference in prevalence, in percentage points (pp), between 2003 and 2023. This approach captures the raw magnitude of change over time. Relative decline was calculated as the percentage reduction from the 2003 baseline prevalence, which reflects the proportional decrease relative to the original level of smoking. Examining both absolute and relative declines allowed us to assess not only how much smoking prevalence dropped in terms of sheer magnitude, but also how substantial the decline was compared to the initial burden. This dual approach provides a more nuanced comparison of trends in smoking prevalence between Black and White adults, highlighting whether groups with higher baseline prevalence experienced comparable proportional reductions or were left behind despite similar absolute drops [42-44]. All available CHIS survey years between 2003 and 2023 were included to generate prevalence estimates. For the decline analysis, only 2003 (baseline) and 2023 (endpoint) values were used to assess 20-year change.

## 4. Results

As shown in Table 1, White adults experienced a larger decline in current smoking than Black adults, both in absolute terms (12.0 vs. 10.9 percentage points) and relative terms (approximately 70% vs. 55%). This pattern suggests that, over this period in California, Black adults may have been more “left behind” in the overall progress of tobacco control and prevention. A similar pattern emerged for flavored tobacco use. White adults showed a larger absolute decline (3.3 vs. 1.1 percentage points), and the relative difference was even greater; the difference was about a 41% decline among White adults compared with only 9% among Black adults. These findings suggest that flavored tobacco control policies in California may have been less effective for Black populations, potentially contributing to widening disparities and leaving them further behind compared to their White counterparts.

**Figure 1.**
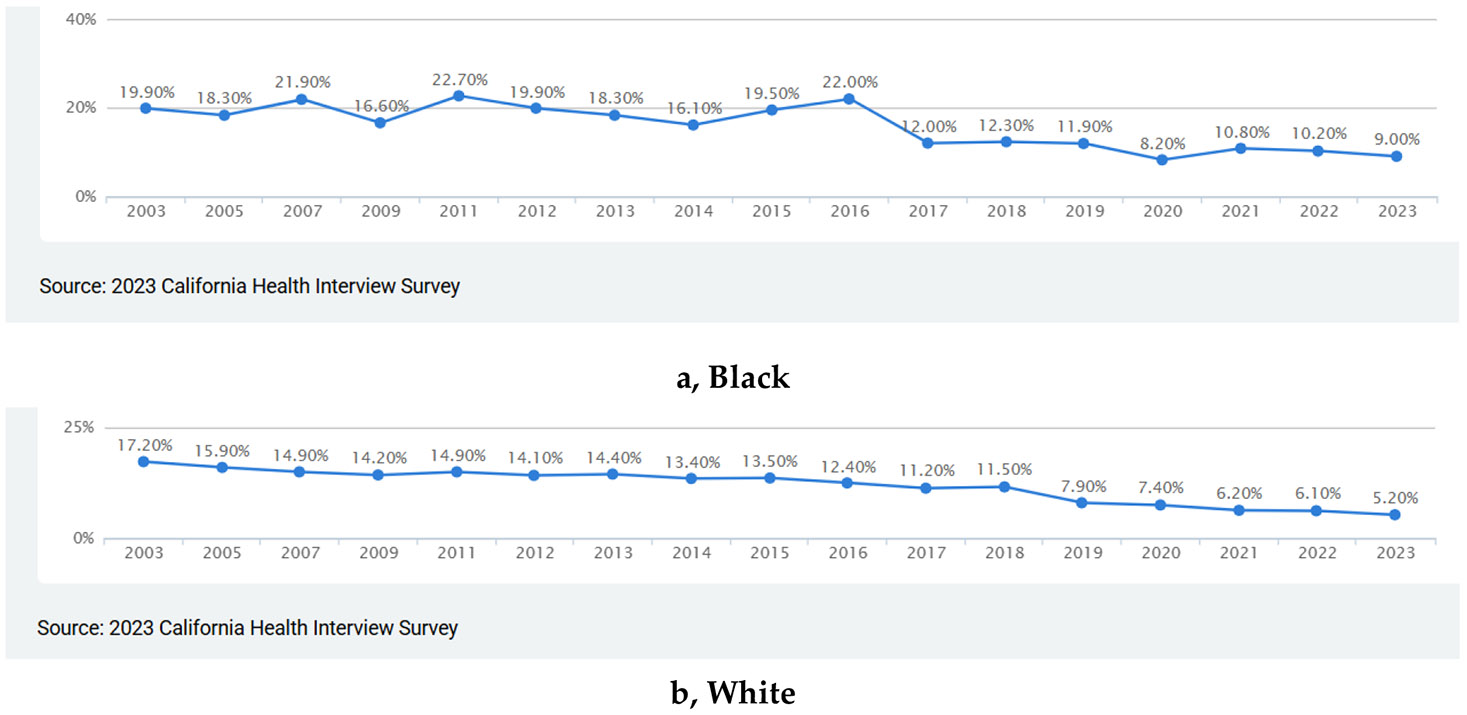
Two decades of trends in current smoking among non-Latino White adults in California, using.

Figure 1a shows two decades of trends in current smoking among non-Latino Black or African American adults in California, based on data from the CHIS. The lower blue line represents the percentage of adults who reported being current smokers, while the upper black line represents those who reported not currently smoking. In 2003, about 19.9% of Black adults reported current smoking. Over the following two decades, this prevalence fluctuated slightly, with modest peaks around 21.9% in 2007 and 22.7% in 2010, followed by gradual declines thereafter. By 2023, the prevalence had dropped to 9.0%, marking an overall absolute decline of 10.9 percentage points and a relative decline of about 55%. Meanwhile, the proportion of non-smokers rose steadily from 80.1% in 2003 to 91.0% in 2023. This gradual increase mirrors the overall success of tobacco control efforts, although the earlier years show some instability, with dips in non-smoking prevalence coinciding with small upticks in smoking rates (e.g., 2007, 2010, 2015). These trends suggest progress in reducing smoking among Black adults in California, but the pace of decline appears modest and uneven over time. The data imply that while tobacco control policies have contributed to overall reductions, they may not have uniformly or consistently reached Black communities, which aligns with broader evidence of persistent racial gaps in tobacco control gains.

Figure 1b depicts two decades of trends in current smoking among non-Latino White adults in California, using data from the California Health Interview Survey (CHIS). The lower blue line represents the percentage of adults who reported being current smokers, while the upper black line shows those who reported not currently smoking. In 2003, 17.2% of White adults reported current smoking. This rate declined steadily and fairly consistently across the following two decades, with only minor fluctuations. By 2023, the prevalence had fallen to 5.2%, representing an absolute decline of 12.0 percentage points and a relative decline of approximately 70%. This pattern reflects a much sharper and more sustained reduction than what was observed among Black adults during the same period. Conversely, the proportion of non-smokers among White adults rose from 82.8% in 2003 to 92.6% in 2023. The upward trend in non-smoking was relatively stable and continuous, with only small temporary dips in the mid-2000s and around 2018. Overall, these data show that tobacco control efforts in California have been highly effective for White populations, producing a large and sustained reduction in smoking. When considered alongside the more modest decline among Black adults, this pattern points to widening racial disparities in the benefits of tobacco control over time, suggesting that existing policies may have had uneven reach or impact across racial groups.

**Figure 2.**
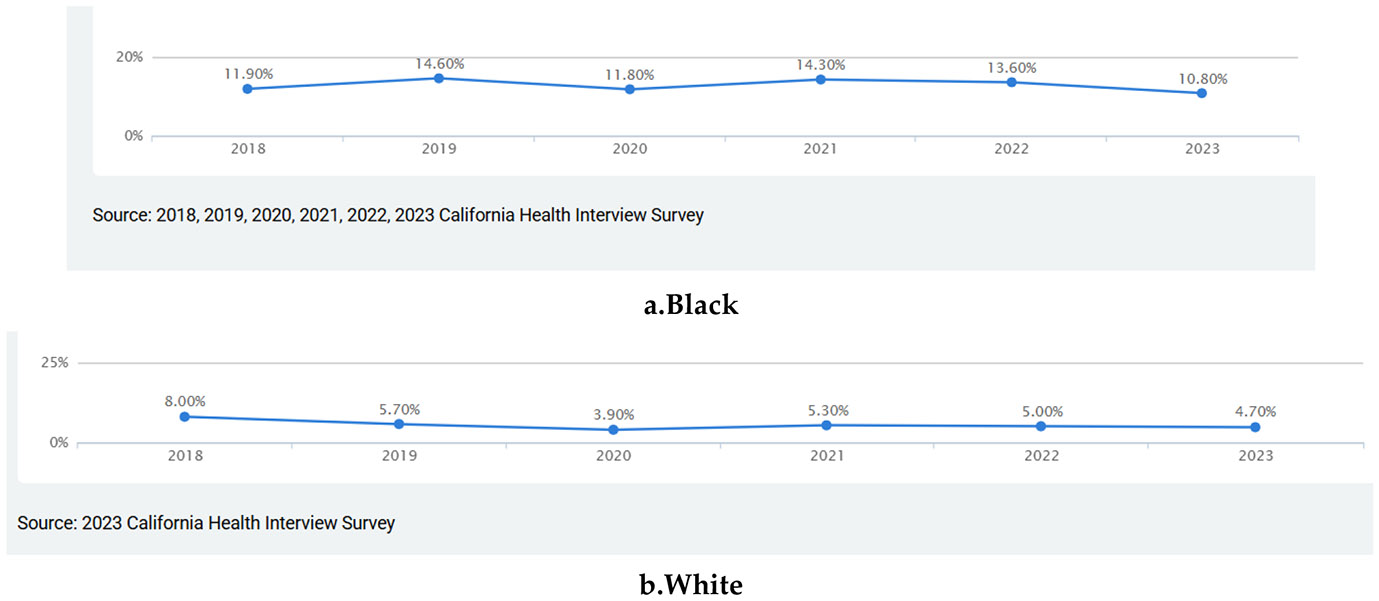
Two decades of trends in current use of flavored tobacco products among non-Latino White adults in California, using.

Figure 2a shows the prevalence of current (past 30 days) flavored tobacco product (FTP) use among Black or African American adults in California, based on data from the California Health Interview Survey (CHIS). The blue line represents those who reported current FTP use, while the black line shows those who reported not using flavored tobacco products. Between 2018 and 2023, the proportion of Black adults using flavored tobacco products remained relatively stable, with only modest year-to-year variation. In 2018, 11.9% reported current use, followed by 14.6% in 2019, 11.8% in 2020, 14.3% in 2021, 13.6% in 2022, and 10.8% in 2023. Across this six-year period, the absolute decline was only about 1.1 percentage points (from 11.9% to 10.8%), representing a relative reduction of less than 10%. In contrast to the substantial and steady declines observed among White adults over the same timeframe, this pattern suggests that flavored tobacco control policies in California have had far less impact on reducing flavored product use among Black adults. The persistently high levels of flavored tobacco use highlight potential gaps in the reach, enforcement, or cultural relevance of existing policies and interventions for Black communities.

Figure 2b displays the prevalence of current (past 30 days) flavored tobacco product (FTP) use among non-Latino White adults in California, based on data from the California Health Interview Survey (CHIS). The blue line represents those who reported current FTP use, and the black line represents those who reported not using flavored tobacco products. Between 2018 and 2023, FTP use among White adults declined notably and fairly consistently. In 2018, 8.0% of White adults reported current use of flavored tobacco products. This prevalence decreased to 5.7% in 2019, 3.9% in 2020, 5.3% in 2021, 5.0% in 2022, and 4.7% in 2023. Overall, this represents an absolute decline of 3.3 percentage points and a relative reduction of about 41%. During the same period, the proportion of White adults not using flavored products rose from 92.0% to 95.3%. This steady upward trend suggests that California’s flavored tobacco restrictions and prevention efforts have been relatively effective for White populations, contributing to a gradual reduction in flavored product use over time. When contrasted with the minimal decline among Black adults over the same years, these findings suggest that flavored tobacco control policies have had a stronger impact on reducing use among White adults, potentially widening racial disparities in flavored product use.

## 5. Discussion

In this analysis of CHIS data from 2003–2023, we found that smoking declined among both Black and White adults in California. However, the magnitude of decline was not equal. White adults experienced larger reductions in both absolute and relative terms than Black adults. For current smoking, prevalence dropped by nearly 70% among White adults compared with about 55% among Black adults. The disparities were even more striking for flavored tobacco use, where declines were minimal for Black adults. These results suggest that Black adults may have experienced smaller relative reductions by some of California’s tobacco control progress.

California is not the only state that has examined the effects of flavored tobacco restrictions. A study [45] in Massachusetts investigated how a statewide ban on flavored products, including menthol, influenced tobacco use patterns and whether the impact differed for Black and White users, given the disproportionate targeting of menthol marketing toward Black communities. Researchers conducted an online survey, distributed through both household mailings and a panel provider, in eleven communities with higher-than-average proportions of Black, Indigenous, and other People of Color (“racially and ethnically diverse populations). The study included 63 Black and 231 White non-Hispanic residents who had used menthol or other flavored tobacco within the past year. Survey measures focused on product access, use, and quitting behaviors, and racial differences were analyzed using Pearson chi-square tests. Results showed that more than half of participants (53% of White and 57% of Black respondents) believed the law made obtaining menthol products more difficult. Still, about two-thirds (67% of White and 64% of Black respondents) reported traveling to other states to purchase menthol tobacco. Black users were significantly more likely than White users to obtain menthol products through street sales (P ≤ .05). Approximately one-third of participants (28% of White and 32% of Black respondents) felt the law made quitting easier, and a similar share (27% of White and 34% of Black respondents) reported quitting completely in the past year. The study concluded that flavored tobacco restrictions may promote cessation and could do so equitably across racial groups. However, patterns of cross-border purchases and street sales highlight ongoing access issues and reinforce the importance of national-level policy alongside stronger cessation support. [45]

A study [46] explored racial differences in smoking patterns over time among Black and non-Hispanic White adults in California. Data were drawn from the California Tobacco Survey covering the years 1996 to 2008. Analyses focused on changes in smoking prevalence and cessation across the two groups. Both Black and non-Hispanic White adults showed declines in lifetime and current smoking during the 12-year period. Substantial reductions were observed in the proportion of heavy daily smokers in both groups. Among Black adults, slight increases were seen in light and intermittent smoking as well as moderate daily smoking, while these categories rose more sharply among White adults. Quit success increased among Black smokers and, to a lesser extent, among White smokers. The patterns observed highlight the progress made through California’s tobacco control policies, particularly in reducing heavy smoking. At the same time, the growth in light, intermittent, and moderate daily smoking suggests that cessation strategies should increasingly address lighter smoking behaviors to sustain and expand public health gains. [46]

In 2009, the United States prohibited flavored cigarettes except for menthol, yet other flavored tobacco products (FTPs) remained on the market. Use of FTPs is disproportionately higher among women, racial and ethnic minorities, adolescents, sexual minority groups, and individuals with lower socioeconomic status. In response, several local governments have implemented restrictions on the sale of FTPs, with the potential to reduce disparities if these regulations effectively reach vulnerable populations. A study [47] evaluated the degree to which such restrictions extended to disproportionately affected groups, referred to as “reach equity.” Researchers identified 189 jurisdictions with FTP-related policies in place as of December 31, 2018. These jurisdictions were linked to demographic characteristics such as race/ethnicity, gender, age, partnered same-sex households, and poverty status, and analyses were stratified by the strength of the policies. Reach Ratios (ReRas) were then used to measure whether specific groups were more or less likely to be covered relative to their representation in the U.S. population. Overall, flavor restrictions encompassed 6.3% of the U.S. population (about 20 million people) across seven states, with only 0.9% covered under strong policies (12.7% of the total). Results showed that young adults, women, Hispanics, African Americans, Asians, partnered same-sex households, and individuals living below poverty were more likely to be covered by flavor policies. By contrast, youth, American Indians/Alaska Natives (AIAN), and Native Hawaiians/Pacific Islanders (NHPI) were underrepresented. Stronger policies demonstrated favorable reach equity to young adults, low-income groups, Asians, NHPIs, individuals identifying with two or more races, and partnered same-sex households. However, they were less equitable in reach for women, youth, Hispanics, AIAN, and African Americans. Findings suggest that existing flavor policies provide greater coverage for several, but not all, high-risk groups. Expanding the adoption of stronger, more comprehensive policies is needed to ensure that subgroups most vulnerable to FTP use are adequately protected [47].

For example, the Food and Drug Administration (FDA) and state regulators need evidence on how a ban on characterizing flavors in cigarettes and cigars—products disproportionately used by African American/Black (AA/B) individuals—might influence racial differences in overall tobacco use and flavored product use. While such policies have the potential to reduce tobacco-related harm, little is known about how AA/B individuals who smoke menthol cigarettes might respond to these restrictions or what strategies could help amplify their intended benefits. In a mixed-methods study of AA/B menthol smokers in Richmond, Virginia [48], stronger quit intentions following a hypothetical menthol ban were linked to greater motivation to quit due to information about health hazards and the financial costs of cigarettes. In contrast, individuals who reported concerns about post-cessation weight gain expressed lower quit intentions. Qualitative interviews highlighted several themes that may shape responses to a menthol ban, including smoking for stress relief, perceptions of harm and addiction related to flavored products, trusted sources of cessation information, and varied prior experiences with quitting. Together, these findings suggest that culturally specific cessation efforts—especially those that emphasize the health and financial benefits of quitting and include testimonials from individuals who have successfully quit—might support AA/B menthol smokers in the context of a menthol ban. At the same time, addressing concerns about weight gain and stress management may be critical for maximizing the equity impact of flavor bans. Tailoring interventions to the cultural and structural realities faced by AA/B communities could help ensure that the FDA’s proposed flavor restrictions contribute to reducing, rather than inadvertently widening, racial disparities in tobacco-related outcomes [48].

California has made several efforts to reduce tobacco use within Black communities [46], often by targeting the structural barriers and industry tactics that sustain disparities [49]. For over 30 years, the California Tobacco Control Program (CTCP) [50] has used state-funded strategies, drawing from Proposition 99 (1988) and Proposition 56 (2016), to support community and school-based education, media campaigns, and local health initiatives. For years, due to CTCP, it was said that welcome to California. America's Largest Non-Smoking Section [50]. In recent years, CDPH has released targeted grants under initiatives such as *Ending California’s Tobacco Epidemic in Every Community*, explicitly prioritizing African American/Black populations among others, and specific RFAs have supported regional programs aimed at Black populations [51]. Local campaigns—like Los Angeles County’s “Done with Menthol”—have tailored messaging to communities of color, using persuasive creative strategies and successfully increasing engagement with cessation resources like quitlines and LAQuits [52]. On the policy front, statewide laws such as Senate Bill 793 and Proposition 31 (2022), which ban most flavored tobacco products, including menthol, help limit the availability of products that disproportionately affect Black smokers. Altogether, while the evidence suggests these efforts may help weaken tobacco’s grip—particularly through reducing access to menthol products and elevating quitting support—persistent disparities remind us that continued, culturally responsive support and sustained funding are still needed to advance equitable outcomes [53,54].

Our findings are consistent with national reports showing uneven declines in smoking across racial and ethnic groups [33,55-57]. Nationally, smoking prevalence remains higher in some marginalized populations [58-60], and menthol use—disproportionately common among Black smokers [61-63]—has slowed declines in cessation and contributed to persistent disparities. International studies also demonstrate similar inequities, such as in New Zealand, where smoking prevalence has decreased overall but remains disproportionately high among Māori and Pacific peoples [15,64,65]. These patterns suggest that unequal progress is not unique to California, but part of a broader structural issue where tobacco control gains accrue more to socially advantaged groups [66].

One possible explanation for the slower decline in tobacco use among Black adults is the combination of lower levels of tobacco harm knowledge and different perceptions of risk, which may weaken motivation to quit. In addition, Black communities often face greater density of tobacco retailers [67], resulting in easier access to products [68,69]. This is compounded by targeted industry marketing, including disproportionate exposure to price promotions [70,71], coupons [72-74], and advertising [75] for menthol and flavored products. Together, these factors create an environment in which initiation is easier, quitting is more difficult, and disparities in tobacco use are sustained over time.

One explanation for the slower decline in tobacco use among Black adults is the long-standing targeted marketing of menthol cigarettes [76,77]. For decades, the tobacco industry has concentrated menthol advertising in predominantly Black communities, often using culturally tailored messages, music, and sponsorships [76,78-80]. This saturation has contributed to the sustained high levels of flavored tobacco use observed among Black adults [63,81]. Menthol itself increases dependence and makes quitting more difficult, creating a unique barrier to cessation that disproportionately affects Black smokers [82,83].

Menthol tobacco products tend to be more addictive than non-menthol varieties [84-86]. The cooling effect of menthol reduces the harshness of smoke or vapor, making it easier to start and harder to quit. This is particularly important because menthol slows nicotine metabolism and can enhance nicotine’s reinforcing effects, which contributes to greater dependence. Because a higher proportion of Black adults use menthol cigarettes compared with White adults, this pattern may contribute to tobacco becoming a more “sticky” behavior within Black communities. In other words, once initiated, menthol use is more likely to persist and quitting becomes more challenging. This dynamic helps explain why Black smokers, despite having similar or even stronger intentions to quit compared with White smokers, often experience lower quit success rates.

Another factor relates to the reach and enforcement of tobacco control policies [87]. While California has implemented some of the most restrictive tobacco laws in the nation, including a statewide flavored tobacco ban, such policies may not have been enforced equally across all neighborhoods [88]. Black communities, often characterized by a higher density of tobacco retailers, may experience weaker implementation and oversight, reducing the effectiveness of these measures. Moreover, public health campaigns may have had lower visibility and cultural resonance among Black populations, limiting their impact.

Disparities in access to cessation resources also play an important role. Black smokers report similar or even higher motivation to quit compared with White smokers, yet they achieve fewer successful quit attempts [58,89-91]. Structural barriers—such as limited healthcare access, lower availability of cessation aids, and fewer culturally tailored programs—contribute to this gap [92]. In addition, mistrust of healthcare institutions may reduce engagement with available services, further undermining the effectiveness of cessation support in Black communities.

The broader social context also influences tobacco use patterns. Higher levels of poverty, chronic stress, and competing needs may limit the capacity of Black adults to prioritize cessation [93]. Mental health challenges and greater exposure to social and environmental stressors, including discrimination and neighborhood disadvantage, may reinforce tobacco use as a coping strategy. These structural inequities mean that even when policies and cessation resources are available, their impact may be diminished by competing demands on individuals and families.

Finally, the framework of Minorities’ Diminished Returns (MDRs) [94] offers an additional explanation. MDRs suggest that socioeconomic resources such as education, income, or health-promoting policies yield smaller health benefits for minoritized groups compared with Whites. If we conceptualize tobacco control policies themselves as a resource, then their “returns” may be weaker for Black adults. This diminished impact may stem from lower reach of interventions, weaker enforcement in disadvantaged communities, or structural barriers that reduce their effectiveness. As a result, despite overall declines in tobacco use in California, Black adults may continue to experience slower or smaller reductions compared with White adults.

### 5.1. Policy and Public Health Implications

These disparities highlight the need for equity-focused approaches to tobacco control. California’s flavored tobacco ban represents an important step, but ongoing evaluation should ensure it reduces flavored product use in populations with the highest burden. Targeted outreach, culturally responsive cessation programs, and equitable enforcement may help close these gaps. At a national level, the delayed implementation of a federal menthol ban continues to limit progress in addressing racial inequities in tobacco use.

### 5.2. Limitations

This study has several limitations. First, the analysis was descriptive and did not adjust for potential confounders such as socioeconomic status or neighborhood context. Second, CHIS data have relied on self-report, which may underestimate smoking prevalence. Third, we focused on Black and White adults only, excluding other racial/ethnic groups that may also face inequities. Finally, trend comparisons used 2003 and 2023 as endpoints, which may oversimplify more nuanced year-to-year variations.

## 6. Conclusions

Despite overall progress in reducing tobacco use in California, declines have been less pronounced among Black adults, particularly for flavored tobacco. These findings reinforce concerns that tobacco control has not advanced equity to the same extent as overall prevalence reduction. Stronger equity-driven policies, combined with targeted community engagement and culturally tailored interventions, may be necessary to ensure that all populations benefit equally from California’s endgame tobacco control efforts.

## Figures and Tables

**Table 1.** Absolute and relative decline in.

Group	Year		Absolute decline	Percent decline
	2003 to	2023		
Current Smoking				
Black	19.9%	9.0%	10.9%	54.8%
White	17.2%	5.2%	12.0%	69.8%
	2018	2023		
Current Flavored Product Use				
White	8.0%	4.7%	3.3%	41.3%
Black	11.9%	10.8%	1.1%	9.2%
